# BBX24 Interacts with DELLA to Regulate UV-B-Induced Photomorphogenesis in *Arabidopsis thaliana*

**DOI:** 10.3390/ijms23137386

**Published:** 2022-07-02

**Authors:** Yuewei Huang, Hui Xiong, Yuxin Xie, Suihua Lyu, Tingting Miao, Tingting Li, Guizhen Lyu, Shaoshan Li

**Affiliations:** 1 Key Laboratory of Ecology and Environmental Science in Guangdong Higher Education, School of Life Science, South China Normal University, Guangzhou 510631, China; huangyueweibs@163.com (Y.H.); xionghui_1995@163.com (H.X.); 15918779581@163.com (Y.X.); 17835204150@163.com (S.L.); 2019022507@m.scnu.edu.cn (T.M.); christinzhen@163.com (G.L.); 2 Guangdong Provincial Key Laboratory of Biotechnology for Plant Development, School of Life Science, South China Normal University, Guangzhou 510631, China; litingtingfc@126.com

**Keywords:** UV-B, DELLA, gibberellins (GA), BBX24, photomorphogenesis, *Arabidopsis thaliana*, hypocotyl inhibition

## Abstract

UV-B radiation, sensed by the photoreceptor UVR8, induces signal transduction for plant photomorphogenesis. UV-B radiation affects the concentration of the endogenous plant hormone gibberellin (GA), which in turn triggers DELLA protein degradation through the 26S proteasome pathway. DELLA is a negative regulator in GA signaling, partially relieving the inhibition of hypocotyl growth induced by UV-B in *Arabidopsis* *thaliana*. However, GAs do usually not work independently but integrate in complex networks linking to other plant hormones and responses to external environmental signals. Until now, our understanding of the regulatory network underlying GA-involved UV-B photomorphogenesis had remained elusive. In the present research, we investigate the crosstalk between the GA and UV-B signaling pathways in UV-B-induced photomorphogenesis of *Arabidopsis thaliana*. Compared with wild type *Landsberg erecta* (*Ler*), the abundance of *HY5*, *CHS*, *FLS,* and *UF3GT* were found to be down-regulated in *rga-24* and *gai-t6* mutants under UV-B radiation, indicating that DELLA is a positive regulator in UV-B-induced photomorphogenesis. Our results indicate that BBX24 interacts with RGA (one of the functional DELLA family members). Furthermore, we also found that RGA interacts with HY5 (the master regulator in plant photomorphogenesis). Collectively, our findings suggest that the HY5–BBX24–DELLA module serves as an important signal regulating network, in which GA is involved in UV-B signaling to regulate hypocotyl inhibition.

## 1. Introduction

Ultraviolet-B (UV-B, 280–315 nm) light is an intrinsic part of the sunlight signal that has significant effects on plant growth and environmental acclimation responses. Numerous studies have demonstrated that UV-B stimulates plant photomorphogenesis, including inhibition of hypocotyl elongation and flavonoid and anthocyanin accumulation, as well as inducing the expression of UV-B-responsive genes [1,2,3]. Plants sense the UV-B signal via UVR8. Upon receiving the UV-B signal, UVR8 (UV RESISTANCE LOCUS 8) depolymerizes from the inactivated dimer into an active monomer form and then shuttles to the nucleus in an active and monomeric form [4,5]. UVR8 re-dimerization is promoted by RUPs (REPRESSOR OF UV-B PHOTOMORPHOGENESIS). RUPs may physically interact with UVR8 and mediate UVR8 re-dimerization, hence negatively regulating UV-B signal transduction [6].

A key mechanism underlying photoreceptor signal transduction has been identified as the direct interaction between photoreceptors and their respective target proteins. Apart from RUPs, UVR8 also physically interacts with COP1, WRKY36, BES1, and BIM1 in the UV-B light signal pathway [7,8,9,10,11,12].

Plant hormones are essential for coordinating the response to environmental stimuli in plants. The plant hormone GA is also involved in regulating seedling morphogenesis. Previous studies have shown that auxin, gibberellins (GAs), and brassinosteroid (BR) promote cell elongation, and in the case of GAs, hypocotyl growth [13,14,15,16]. In particular, in the GA pathway, when endogenous GA appears at a high level, it leads to degradation of DELLA, thereby releasing its inhibitory effect in GA signaling and promoting cell elongation. The canonical process involves GID1 (GA-INSENSITIVE DWARF MUTANT 1), the receptor of GA, combining with the DELLA protein to form a trimer. The above indicates that the phytohormone GA may act as the mobile cue that is transported from the site of flowering to the site of hypocotyl elongation. While some researchers raise an argument that there exists a noncanonical pathway to destabilize DELLA protein relying on COP1, warmth, and shade [17], others suggest that when endogenous GA is relatively low, DELLA accumulates and inactivates PIFs (PHYTOCHROME INTERACTING FACTORS) to inhibit hypocotyl growth [18,19]. DELLA serves as a negative regulator in the gibberellin signal transduction pathway as well as growth inhibitory proteins of the plant-specific family. The *Arabidopsis* genome encodes five DELLA members, including RGA (REPRESSOR of gal-3), GAI (GA INSENSITIVE), RGL1 (RGA-LIKE1), RGL2 (RGA-LIKE2), and RGL3 (RGA-LIKE3) [20,21]. Among the five DELLA members, GAI and RGA were proved to be the master repressors regulating etiolated growth [22]. It was reported that UV-B inhibited the endogenous GA content by up-regulating GA2ox1 expression, so it is involved in plant morphogenesis [23].

The molecular mechanism of DELLA regulation of transcription relies on the physical interaction with transcription factors, including BBX24, which is a negative regulator of UV-B-induced photomorphogenesis [20]. BBX24 and PIF4 (PHYTOCHROME INTERACTING FACTOR 4) compete with DELLA proteins, thereby advancing the expression of downstream shade-response genes under shade [24]. There are pieces of evidence showing that DELLA and BBX1 are jointly involved in the regulation of long-day flowering in *Arabidopsis*. BBX1 and BBX24 belong to the BBX family and have similar protein structures [24,25,26]. In addition, DELLA interacts with the CO (CONSTANCE) protein, which is structurally similar to the BBX24 protein in relation to high motif similarity and participates in the regulation of flowering under long day length [27].

Our previous research found that under UV-B radiation, BBX24 attenuates UV-B-induced HY5 accumulation and suppresses its transcription activation activity to inhibit photomorphogenesis by interacting with HY5 and is a part of the negative feedback regulation mechanism in the UV-B pathway [25]. HY5-dependent modulation of expression of downstream genes is related to light and GA [28]. Additionally, HY5 acts upstream of DELLA in shade avoidance [23], and GA was significantly inhibited by low temperature in an HY5/HYH-dependent manner [29], so that HY5 could regulate the accumulation of DELLA protein [30].

On the basis of the above background, in the present study we asked whether the BBX24–DELLA module is functional, how the module works, and what is the relationship among HY5–BBX24–DELLA in regulating plant photomorphogenesis. We hypothesize that *Arabidopsis thaliana* UV-B photomorphogenesis relies on the BBX24–DELLA regulatory module, HY5 is key transcription factor in UV-B photomorphogenesis, and BBX24 also interacts with HY5; thus, the HY5–BBX24–DELLA module would affect and associate with the crosstalk of the UV-B response as well as GA. Here, we use *rga-24*, *rga-24gai-t6*, *gai-t6*, and *rga-24bbx24* mutants to elucidate the mechanism of the BBX24–DELLA module involved in the regulation of UV-B-induced hypocotyl growth inhibition in *Arabidopsis thaliana*. This study also used in vitro and in vivo experiments to confirm that both BBX24 and HY5 interact with RGA (the functional member in the DELLA family). A possible working model of these three factors in the UV-B signaling pathway is proposed.

## 2. Results

### 2.1. UV-B Regulates the Abundance of DELLA Protein

**Figure 1:**
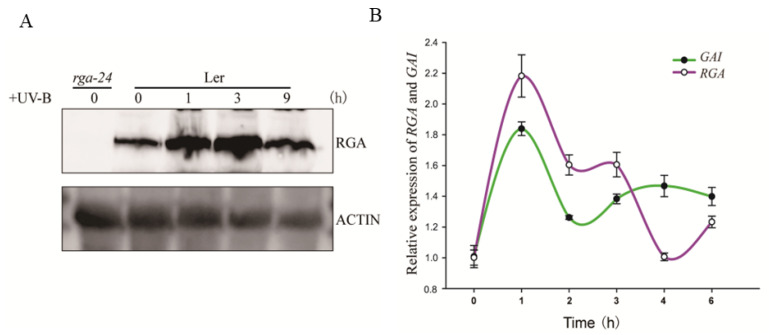
UV-B promotes DELLA accumulation by inducing GA inactivation. (**A**) Wild type *(Ler*) was exposed to UV-B radiation of 0.6 W/m^2^ for 0 h, 1 h, 3 h, and 9 h after growing under white light for 7 days, and then the content of RGA protein was measured. Actin is shown as a protein-loading control. (**B**) The expression of *RGA* and *GAI* under different periods of UV-B irradiation (irradiation intensity is the same as with (**A**)). Ler was transferred to UV-B radiation for 0 h, 1 h, 2 h, 3 h, 4 h, and 6 h after growing under white light for 6 days. Error bars represent the SE of three biological replicates.

To reveal the regulatory network of DELLA in the UV-B signaling pathway, we explored how the abundance of RGA protein changed over time under UV-B radiation. The obtained results showed that the abundance of RGA protein increased upon UV-B radiation, reached the highest level after 3 h of UV-B radiation, and then returned to the basal level after 9 h of UV-B radiation (Figure 1A). Moreover, after 3 h of UV-B-induced radiation, the expression of RGA GAI at the transcription level decreased (Figure 1B). The two results indicated that the response of the RGA protein upon UV-B radiation might occur in the early stage of the reaction.

### 2.2. DELLA Positively Regulates UV-B-Induced Photomorphogenesis

**Figure 2:**
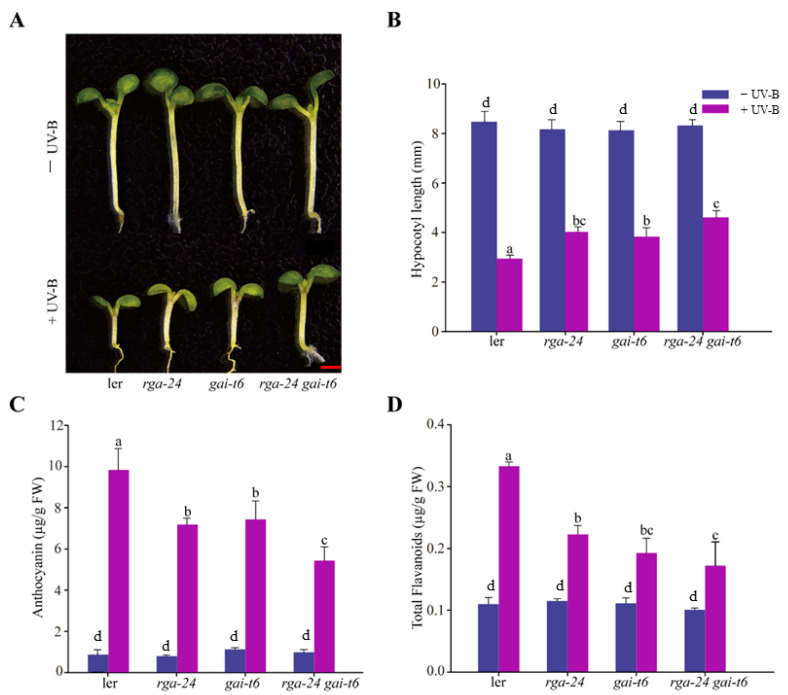
DELLA promotes hypocotyl elongation and the synthesis of flavonoid under UV-B radiation. (**A**) Images of the *Ler rga-24*, *gai-t6*, and *rga-24 gai-t6* seedlings. Bar = 1 mm. (**B**) The hypocotyl lengths of the indicated genotypes. (**C**) Anthocyanin content. (**D**) Flavonoid content. All the above indicated seedlings were transferred to UV-B radiation of 0.6 W/m^2^ for 4 days after growing under white light for 2 days, and then the content of anthocyanin and flavonoid was measured. ‘FW’ indicated fresh weight. Error bars indicate SE (*n* ≥ 3). The letters ‘a’ to ‘c’ indicate statistically significant differences between the indicated seedlings grown in the presence of UV-B radiation, as determined by Tukey’s least significant difference test (*p* < 0.05).

Inhibition of hypocotyl length is one of the typical features of photomorphogenesis. We found that the hypocotyls of the mutants with depletion of DELLA were significantly longer than those of Ler in the presence of UV-B radiation, and the hypocotyl lengths of the *rga-24 gai-t6* mutants were the longest (Figure 2A,B). These results suggested that deletion of the *DELLA* gene in this ecotype alleviated the phenomenon of UV-B-induced hypocotyl elongation inhibition. The anthocyanin and flavonoid contents in the above mutants were significantly lower than those in *Ler* under UV-B radiation. Under the same conditions, the concentration of flavonoids in the *rga-24 gai-t6* double mutant was the lowest, suggesting that DELLA appears to promote the synthesis of flavonoids under UV-B radiation (Figure 2C,D). These results suggested that DELLA is involved in UV-B photomorphogenesis. Additionally, two members of the DELLA family, RGA and GAI, may cooperate in this process.

**Figure 3:**
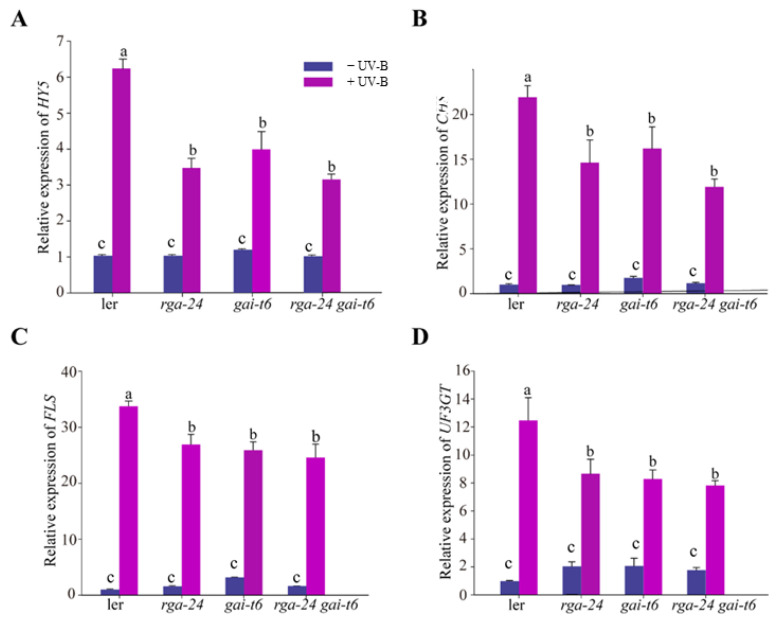
DELLA regulates the gene expression of the UV-B signaling pathway. (**A**–**D**) Quantitative reverse transcription PCR analyses of *HY5*, *CHS*, *FLS,* and *UF3GT* expression, respectively, in the *Ler*, *rga-24*, *gai-t6,* and *rga-24 gai-t6* double mutants. Six-day-old constant white light-grown seedlings were transferred to UV-B for 1 h. The *Actin2* gene was analyzed as an internal control. Error bars represent the SE of three biological replicates. The letters ‘a’ and ‘b’ indicate statistically significant differences between the gene transcription level of the indicated seedlings grown in the presence of UV-B radiation, “c” represents no significant difference between these columns, as determined by Tukey’s least significant difference test (*p* < 0.05).

To further investigate the regulatory mechanism of DELLA in the UV-B signal pathway, the transcription level of the UV-B response gene *HY5* and the flavonoid synthesis pathway regulation genes *CHS*, *FLS*, *UF3GT* in each ecotype were measured by qRT-PCR analysis. The obtained results showed that under UV-B exposure, the transcription levels of *HY5*, *CHS*, *FLS*, and *UF3GT* genes were significantly and substantially up-regulated in each genotype. In addition, the transcription level of *HY5*, *CHS*, *FLS*, and *UF3GT* genes in the *della* mutants was significantly down-regulated compared with that of wild type (*Ler*) (Figure 3). Taken together, DELLA is a positive regulator of UV-B photomorphogenesis.

### 2.3. Antagonistic Regulation of DELLA and BBX24 in UV-B Photomorphogenesis

**Figure 4:**
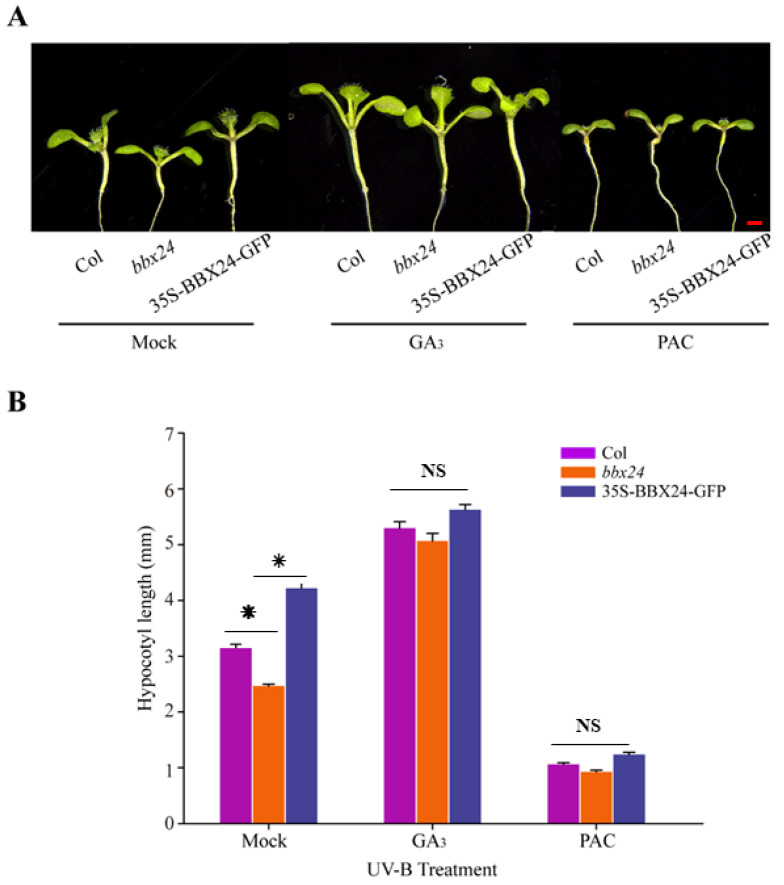
Gibberellins restore hypocotyl length inhibition of BBX24 seedlings under UV-B radiation. (**A**,**B**) Hypocotyl phenotypic analysis. Seedlings of *Col-o*, *bbx24* mutant, and 35S-BBX24-GFP were grown for 5 days and then transferred to UV-B radiation after growth for 3 days under white light. Images of the representative seedlings (mock, 1/2MS; GA_3_: 5 μM PAC: 0.5 μM, GA synthesis inhibitor) are shown in (**A**). The hypocotyl lengths of the indicated genotypes were measured and are shown in (**B**). Error bars indicate SE (*n* ≥ 15). Bar = 1 mm. The asterisks indicate statistically significant differences between the hypocotyl lengths of the indicated seedlings grown in the presence of UV-B radiation, “NS” means “No Significance” represents no significant difference between these columns, as determined by Tukey’s least significant difference test (*p* < 0.05).

DELLA and BBX24 had been reported to interact with each other in the pathway of shade avoidance. Therefore, we are motivated to further explore whether DELLA and BBX24 are jointly involved in the regulation of UV-B photomorphogenesis. Our results showed that in the presence of UV-B radiation, the hypocotyl length of *bbx24* was significantly shorter than that of wild type *Columbia-o* (*Col-o*), and the hypocotyl length of the ecotype of 35S-BBX24-GFP was longer than that of *Col-o*, which is consistent with the conclusion that BBX24 is a negative regulator of UV-B photomorphogenesis (Figure 4). In the medium supplemented with GA_3_ (5 μM) under UV-B radiation, there was no significant difference in the length of the hypocotyl among these ecotypes (Figure 4), suggesting that GA_3_ restored the inhibition of the hypocotyl in the *bbx24* mutant. In the medium supplemented with 0.5 μM PAC (an inhibitor of gibberellin acid biosynthesis), the hypocotyl elongation of *Col-o*, *bbx24*, and 35S-BBX24-GFP was substantially inhibited, and the length of the hypocotyl among these treated groups was not significantly difference under UV-B radiation (Figure 4B). This suggests that GA_3_ relieved the *Arabidopsis* hypocotyl inhibition of *bbx24* during UV-B photomorphogenesis. These results suggested that DELLA is the link between GA and UV-B photomorphogenesis, which co-regulates photomorphogenesis of *Arabidopsis* with BBX24.

**Figure 5:**
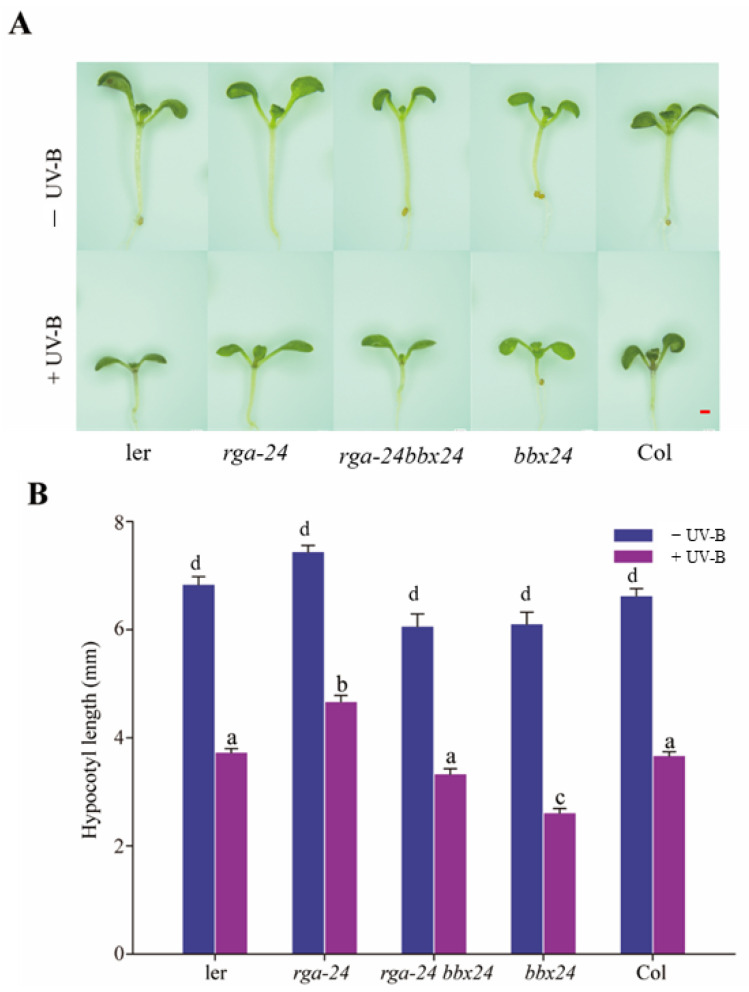
DELLA and BBX24 have antagonistic effects on the UV-B signal transduction pathway. (**A**, **B**) Hypocotyl phenotypic analysis. Seedlings of wild type (*Ler* and *Col-o*), *rga-24*, *bbx24*, and *rga-24 bbx24* double mutant were grown for 8 days in the presence of UV-B radiation. Images of the representative seedlings are shown in (**A**). The hypocotyl lengths of the indicated genotypes were measured and are shown in (**B**). Bar = 1 mm. Error bars indicate SE (*n* ≥ 15). The letters ‘a’ to ‘c’ indicate statistically significant differences between the hypocotyl lengths of the indicated seedlings grown in the presence of UV-B radiation, as determined by Tukey’s least significant difference test (*p* < 0.05).

When exposed to UV-B radiation, inhibition of the *rga-24* mutant hypocotyl was the weakest, and *bbx24* mutant hypocotyl inhibition was the strongest. The *rga-24 bbx24* double mutant’s hypocotyl length was between that of the *rga-24* and *bbx24* mutants (Figure 5). These results showed that RGA and BBX24 antagonistically regulate UV-B photomorphogenesis.

### 2.4. RGA Physically Interacts with BBX24 Protein to Regulate UV-B Signaling

**Figure 6:**
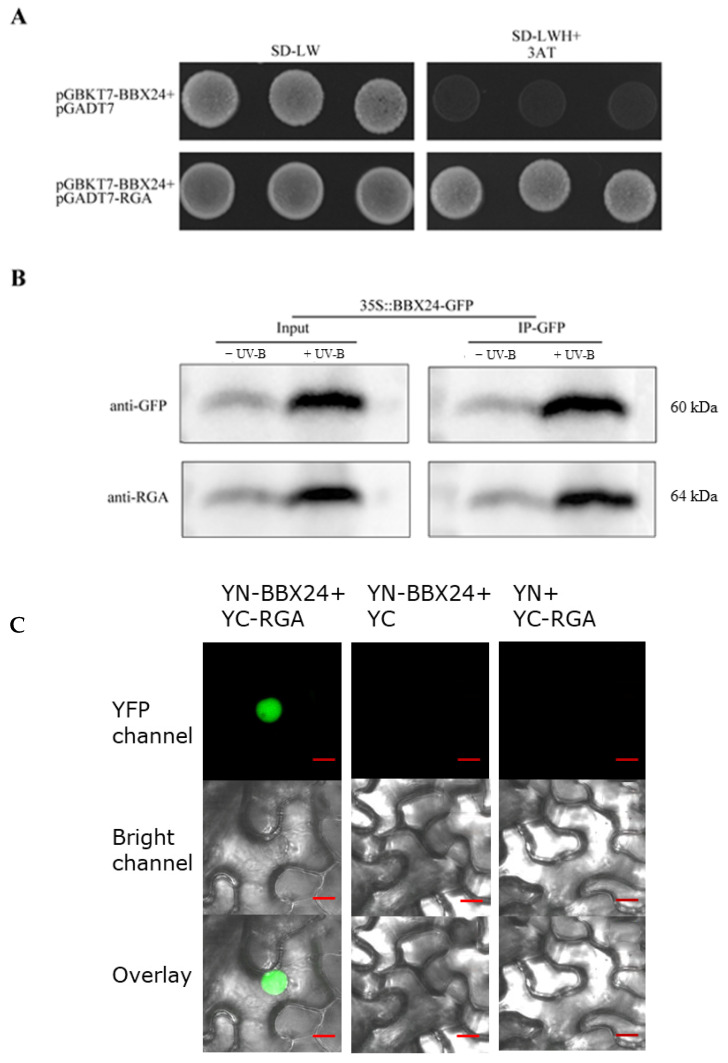
RGA physically interacts with BBX24. (**A**) Yeast was transformed with a bait and a prey construct. The bait vector constructs contain the GAL4-DNA binding domain fused with BBX24, and the prey vector constructs contain the GAL4-activation domain fused with RGA in the LexA system. Transformants were grown on SD/-Leu/-Trp/-Ade/-His (+X-α-gal) selective media. pGBKT7-BBX24/pGADT7-RGA was used as the positive control. (**B**) Co-immunoprecipitation assay using 7-day-old transgenic seedlings expressing 35S-BBX24-GFP treated with or without UV-B radiation for 6 h after treating with UV-B radiation for 10 h. Input: immunoblots showing the level of BBX24-GFP and RGA in the total protein extract. IP-GFP: immunoprecipitation products precipitated by the anti-GFP antibody. Total proteins (input) or immunoprecipitation products were probed in immunoblots with antibodies to GFP or RGA. (**C**) A BiFC assay was used to reconfirm the interactions between BBX24 and RGA. Full-length BBX24 and RGA were fused to the split of N- or C-terminal (YFP-N or YFP-C) fragments of YFP. Unfused YFP N-terminal (YFP-N) and YFP C-terminal (YFP-C) were used as negative controls. Merge, merged images (overlay) of the YFP channel and bright field channel. Scale bars: 10 μm. Fluorescence signal was detected by confocal microscopy.

To further investigate the molecular mechanism between RGA and BBX24, we set out to test the possible interactions between RGA and BBX24 by using the Yeast Two Hybrid (Y2H) assay. Firstly, the target protein, positive control, negative control, and the interacting proteins were co-transformed into the yeast medium DDO (SD/-Leu-Trp), and colony growth was observed. Then the yeast rotation experiment of the interacting proteins was verified on the yeast medium QDO (SD/-Leu-Trp-His-Ade), with pGADT7 empty vector used as a negative control. The interaction between BBX24 and RGA was observed in 10^−1^, 10^−2^, and 10^−3^ dilutions of yeast liquid. In addition, the interaction between RGA and BBX24 was verified by immunoprecipitation experiments, with and without the presence of UV-B radiation. Our obtained results demonstrated that RGA could directly interact with BBX24, whose interaction was also enhanced by the UV-B radiation (Figure 6A,B). To examine the subcellular locations where RGA interacts with BBX24, we next conducted a Bimolecular Fluorescent Complimentary (BiFC) assay using *N. benthamiana* leaves. We clearly detected a strong nuclear localized YFP signal when transiently co-expressing both YFP-N-BBX24(YN-BBX24) and YFP-C-RGA(YC-RGA) as demonstrated in Figure 6C. However, the negative controls (YFP-N-BBX24 and YFP-C as well as YFP-N and YFP-C-RGA) did not produce a YFP signal (Figure 6C). Taken together, these data suggest that BBX24 physically interacts with RGA, and the BBX24-RGA molecular module is involved in UV-B signaling.

### 2.5. HY5 Physically Interacts with RGA Protein

**Figure 7:**
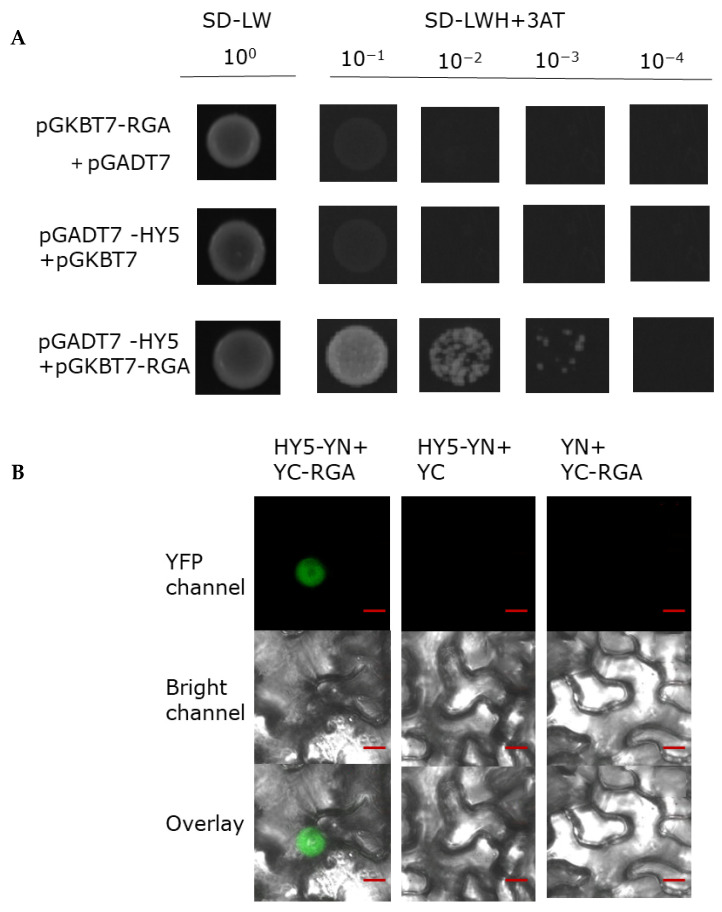
RGA physically interacts with HY5. (**A**) Yeast was transformed with a ‘bait and a prey’ construct. The bait vector constructs contain a GAL4-DNA binding domain fused with RGA, and the prey vector constructs contain the GAL4-activation domain fused with HY5 in the LexA system. Transformants were grown on SD/-Leu/-Trp/-Ade/-His (+X-α-gal) selective media. pGBKT7-RGA/pGADT7-HY5 was used as the positive control. (**B**) A BiFC assay was used to verify the interaction between RGA and HY5. Full-length HY5 and RGA were respectively fused to the split of the N- or C-terminal (YFP-N and YFP-C) fragments of YFP. Unfused YFP-N-terminal (YFP-N) and YFP-C-terminal (YFP-C) were used as negative controls. Overlay, overlay image included YFP channel and bright field channel. Scale bars: 10 μm. Fluorescence signal was detected by confocal microscopy.

HY5 is a master gene in photomorphogenesis and promotes accumulation of DELLA. In the process of verifying the biochemical interaction between RGA and BBX24, we also explored the interaction between RGA and HY5. Interestingly, we found that RGA could also physically interact with HY5 in the yeast two hybrid assay (Figure 7A). To further corroborate the interaction between these two proteins, we next conducted a BiFC assay and found that HY5–YN and YC-RGA, but not the negative controls, could produce an obvious nuclear localized YFP signal. These results suggested that BBX24 is able to interact with RGA.

## 3. Discussion

In our present work, under UV-B radiation, the accumulation of RGA protein of DELLA family members increased significantly in the earlier stage but fell to the basal level after 9 h of UV-B radiation, perhaps because of the GA signal [21], and interacted or inhibited by some transcriptional factors such as MYC2 and PIFs [13,31] so as to help plant growth and development be in a state of dynamic equilibrium (Figure 1A), but there was no significant change in the expression of *RGA* transcription level (Figure 1B). UV-B radiation may not directly regulate *DELLA* expression, but it promotes the accumulation of DELLA protein by inhibiting the endogenous GA content.

The transcription factor DELLA protein connects a collection of environmental signals [16,18,32,33]. The loss-of-function mutant *della* caused excessive plant growth in daylight [13,14,15]. Both DELLA and UV-B can activate the phenylpropane pathway and promote the accumulation of phenolic compounds, such as flavonoids, anthocyanins, and mustard oils, to adapt to environmental changes [2,8,34,35,36,37]. HY5 is the core transcription factor for photomorphogenesis, which regulates nearly one-third of the gene expression in the *Arabidopsis* genome and is also necessary for the UV-B transduction pathway [38]. Under UV-B radiation, the transcriptional up-regulation folds of *HY5* and flavonoid synthesis genes *CHS, FLS,* and *UF3GT* in *rga-24* and *gai-t6* mutants were significantly lower than those of the wild type plants (Figure 3). Therefore, it is suggested that DELLA is a positive regulator of UV-B photomorphogenesis. The accumulation of flavonoids and anthocyanins in the *rga-24 gai-t6* double mutant was also significantly lower than that in the single mutant (Figure 2C,D). The results indicate that RGA and GAI synergistically regulate UV-B photomorphogenesis. In addition, the effects of RGA and GAI on *Arabidopsis* hypocotyl elongation and flavonoid accumulation are not additive (Figure 2), and there is a certain degree of functional redundancy.

Light and gibberellin antagonistically regulate plant morphogenesis [13]. UV-B radiation inhibits the elongation of hypocotyls by regulating endogenous hormone signals, such as brassinosteroids and auxin [39]. In addition, BBX24 and DELLA participate in the regulation of the shade response of *Arabidopsis* [24], and BBX24 is a negative regulator of UV-B photomorphogenesis [25]. Through additional gibberellin application, it was found that gibberellin can restore the phenotype of *bbx24* mutant under UV-B radiation (Figure 4), which implies that the BBX24–DELLA functional hub may be the link between the UV-B signal and the GA transduction signal. The hypocotyl phenotype of the *rga-24 bbx24* mutant under UV-B radiation was similar to the wild type (Figure 5), indicating that RGA and BBX24 are jointly involved in UV-B photomorphogenesis. The results of the Yeast Two Hybridization, co-immunoprecipitation (Co-IP), and biomolecular fluorescence complementation (BiFC) assays showed that there is an interaction between RGA protein and BBX24 protein (Figure 6). The interaction of BBX24 and RGA appeared to predominantly occur under UV-B radiation, and the degree of protein expression was greater than in the group that was untreated with UV-B (Figure 6B). The results also indicated that HY5 interacts with RGA (Figure 7). When the three proteins occur together in a possible module, BBX24 and HY5 may compete in interacting with RGA (Figure 6C and Figure 7B). These results suggest that RGA would work in concert with BBX24 and HY5 proteins to coordinate the control of hypocotyl growth in response to UV-B signals.

**Figure 8:**
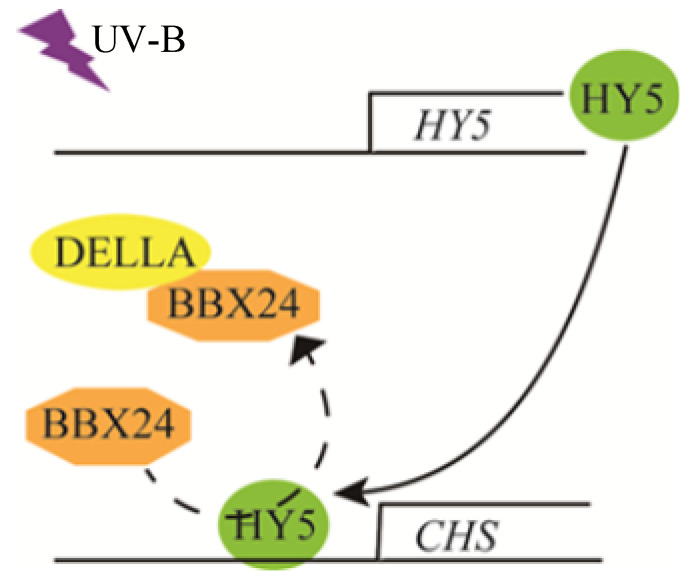
A possible working model of DELLA involved in UV-B-induced photomorphogenesis.

We have revealed that DELLA is a positive regulator of photomorphogenesis, at least a part of which regulates UV-B signal transduction through interaction with the BBX24 protein. These research results provide more possibilities for elucidating the mechanism of UV-B and GA co-regulating plant growth. BBX24 interacts with the core transcription factor HY5 protein to inhibit UV-B photomorphogenesis under UV-B radiation [25]. A UV-B signal is received by the UV-B photoreceptor UVR8. The COP1-UVR8 interaction stabilizes and activates the transcriptional activity of HY5, leading to UV-B-regulated gene expression and photomorphogenesis. The negative regulator BBX24 protein interacts with HY5 to fine-tune the UV-B response. Moreover, HY5 may be involved in the BBX24–DELLA module. The three components together control hypocotyl growth in response to UV-B. However, the function of the DELLA protein in the BBX24-HY5 pathway and whether their interaction happens in the cell nucleus or not still needs further elucidation, particularly in relation to the mechanism of DELLA regulation of UV-B photomorphogenesis. We will keep focusing on whether the components of the HY5–BBX24–DELLA module compete with each other in the UV-B signaling pathway so as to provide new insights into the mechanism of UV-B photomorphogenesis. Figure 8 presents a possible model of the overall process.

## 4. Materials and Methods

### 4.1. Plant Materials and UV-B Treatment

*Arabidopsis thaliana Columbia-o* (*Col-o*) and *Landsberg erecta* (*Ler*) ecotypes were used as the wild types, and the mutants below were mutated in their respective backgrounds.

The transfer DNA insertion mutant *bbx24* (SALK_067473) was in the Col-0 ecotype background. The entire ORF-deleted mutant *rga-24* and DS transposon insertion mutant *gai-t6* were in the *Landsberg erecta* (*Ler*) ecotype background. The *rga-24* *gai-t6* and *rga-24 bbx24* double mutants were prepared by genetic crossing, and their identities were verified by genotyping. The information on primers is listed in Supplemental Table S1, and the 35S-BBX24-GFP overexpression plant has been described previously (Jiang et al., 2012).

Seeds were sterilized with 30% (*v*/*v*) commercial bleach for 10 min, washed with sterile water, then sown on 1/2 Murashige and Skoog (MS) medium containing 0.8% agar and 3% sucrose and stratified for 4 days at 4 °C in the dark before being grown under white light (Philips TLD30W/865 tubes; Quantitherm Light Meter, Hansatech, UK; 100 μmol m^−2^·s^−1^) for several days (light/dark, 16/8 h) at 22 °C. The UV-B treatment was performed by narrowband UV-B tubes (Philips TL 20W/01 RS; 0.6 W m^−2^).

### 4.2. Plant Hypocotyl Measurements

The phenotype of the hypocotyl of the different ecotypes was observed using a fluorescence microscope (M205FA, Leica, Wetzlar, Germany). Measurement of hypocotyl length was recorded using Image J software (National Institutes of Health, Bethesda, MD, USA) For hypocotyl measurements, a minimum of 15 seedlings was measured for each genotype in each condition.

### 4.3. Anthocyanin and Flavonoid Measurement

To measure flavonoid contents, 0.1 g of 7-day-old seedlings exposed to UV-B were quantified as previously reported by [40], and the measurement of anthocyanin content was based on [41]. All experiments were independently performed three times (biological repeats) per treatment with similar results.

### 4.4. RNA Extraction and Real-Time PCR

Total RNAs were extracted using the Trizol method. cDNAs were synthesized from 2 ng of total RNAs using the PrimerScript RT reagent kit with gDNA Eraser (Perfect Real Time) (Takara, Dalian, China) according to the manufacturer’s instructions. Real-time PCR was performed on the QuantStudio 6 Flex Sequence Detection System (Applied Biosystems, Foster City, CA, USA) using Luna^®^ Universal qPCR Master Mix (NEB, Ipswich, MA, USA) following the recommended conditions. Specific primer pairs for each gene were designed using Oligo 5.0 software. The information on primers is listed in Supplemental Table S1.

### 4.5. GA and PAC Treatments

*Arabidopsis thaliana* wild-type Columbia (Col-o) and its mutant *bbx24*, overexpression transgenic plant 35S-BBX24-GFP, were first sown and germinated for 48 h under white light on wet filter paper. Then, seeds were transferred to 1/2 solid Murashige and Skoog (MS) medium supplemented with synthetic hormones. GA_3_ (Sigma-Aldrich, Germany) and PAC (Sigma-Aldrich, Germany) dissolved in ethanol and diluted using 1/2 MS were added to indicated concentrations, or an equivalent amount of ethanol diluted in 1/2 MS was added as a control treatment. Seedlings were then grown for 6 days under UV-B light and collected for hypocotyl analysis.

### 4.6. Confocal Microscopy

Confocal laser scanning microscopy was performed using a Zeiss LSM710 system (Carl Zeiss AG, Jena, Germany). Yellow fluorescent protein (YFP) and green fluorescent protein (GFP) were excited, and emissions were collected at 488–513 nm.

### 4.7. Yeast Two-Hybrid Assay

For verifying the BBX24 and RGA interaction, full-length BBX24 cDNA was cloned into the pGBKT7 bait vector, and full-length RGA cDNA fragments were cloned into the pGADT7 prey vector. For verifying the HY5 and RGA interaction, the full-length of RGA cDNA was cloned into the pGBKT7 bait vector, and full-length HY5 cDNA fragments were cloned into the pGADT7 prey vector. These generated constructs were transformed into the yeast strain AH109 and selected on dropout medium (SD/–Trp/–Leu/–His) following the instructions of the manufacturer (Clontech, Dalian, China). The information on primers is listed in Supplemental Table S2.

### 4.8. Co-IP Assays and Immunoblot Analyses

The Co-IP experiment was carried out to confirm the interaction of BBX24 with DELLA in vivo. Total protein was extracted by IP buffer containing 250 mM Tris, 750 mM NaCl, 5 mM EDTA, pH 7.4, 10% (*v*/*v*) Triton X-100, and 20 μL/mL protease inhibitor cocktail (Sigma-Aldrich, St. Louis, MO, USA) before centrifugation at 12,000× *g* for 10 min and boiling at 95 °C for 10 min. The protein extracts were precipitated with a monoclonal antibody GFP-Trap^®^ MA (KT Life Technology, Shenzhen, China) overnight at 4 °C, and the beads were then washed three times in IP buffer. Total protein and immunoprecipitated samples were analyzed by Western blotting by using anti-RGA (PHYTO AB, San Jose, CA, USA) and anti-GFP (Cell Signaling Technology, Danvers, MA, USA) to detect the BBX24–DELLA interaction.

### 4.9. Bi-Molecular Fluorescence Complementation

The RGA CDS was transferred to the 1300-35s-YFC-gene vector, BBX24 CDS was transferred to 1300-35s-YFN-gene, and HY5 CDS was transferred to the 1300-35s-gene-YFN vector to prepare the targeted plasmid constructs. These generated constructs, namely, 1300-35s-YFC-RGA, 1300-35s-YFN-BBX24, and 1300-35s-HY5–YN were introduced into the *Agrobacterium* strain GV3101, which could carry these targeted constructs growing overnight in Luria–Bertani medium at 28 °C. Cells were pelleted by centrifugation, and the pellet was resuspended in infiltration buffer (10 mM MgCl_2_, 150 mM acetosyringone, and 10 mM MES, pH 5.6) to a final cell density equivalent to OD_600_ = 0.6. We infiltrated the cell suspensions containing the targeted transformant pairs into *N. benthamiana* leaves, then put these treated plants in the dark for 48 h. We detected YFP fluorescence using a confocal laser scanning microscope (LSM710 Meta; Carl Zeiss) after infiltration, which is described above (Section 4.6). The information on primers is listed in Supplemental Materials.

### 4.10. Statistical Analysis

Statistical analyses were carried out using SPSS version 23.0 (IBM) by one-way analysis of variance (ANOVA). Tukey’s least significant difference test was used to deduce statistically significant differences between the mean values indicated in the figure legends (*p* < 0.05), as indicated by letters or symbols in the figures. Figures were prepared using SigmaPlot 12.5 (Systat Software Inc., Chicago, IL, USA).

## Data Availability

The original contributions presented in the study are included in the article/Supplementary Material. Further inquiries can be directed to the corresponding author/s.

## Supplementary Materials

The following supporting information can be downloaded at: https://www.mdpi.com/article/10.3390/ijms23137386/s1.
