# Supplementary material for: BBX24 Interacts with DELLA to Regulate UV-B-Induced Photomorphogenesis in Arabidopsis thaliana
Source: Int J Mol Sci. 2022 Jul 2;23(13):7386. doi: 10.3390/ijms23137386 (PMC9266986; doi:10.3390/ijms23137386)
Supplement: Supplementary file 1 [file ijms-23-07386-s001.zip › ijms-1791128-supplementary.pdf]

Supplementary data

Figure S1. Detection of rga-24 gai-t6 double mutant genotype level. The letter 'M' indicated Maker, 1~17 indicate the number of double mutant.

Figure S2. Identification of rga-24 bbx24 homozygous double mutant A. Detection of rga-24 bbx24 double mutant genotype level. The letter 'M' indicated Maker, 1~3 indicate the number of double mutant. B. Detection of rga-24 bbx24 double mutant gene expression levels. The Actin2 gene was as an internal control Table S1. Primers used for genotyping.

Table S1. Primers used for genotyping and qRT-PCR.

Table S2. Primers used for plasmid constructs.

Supplemental Data

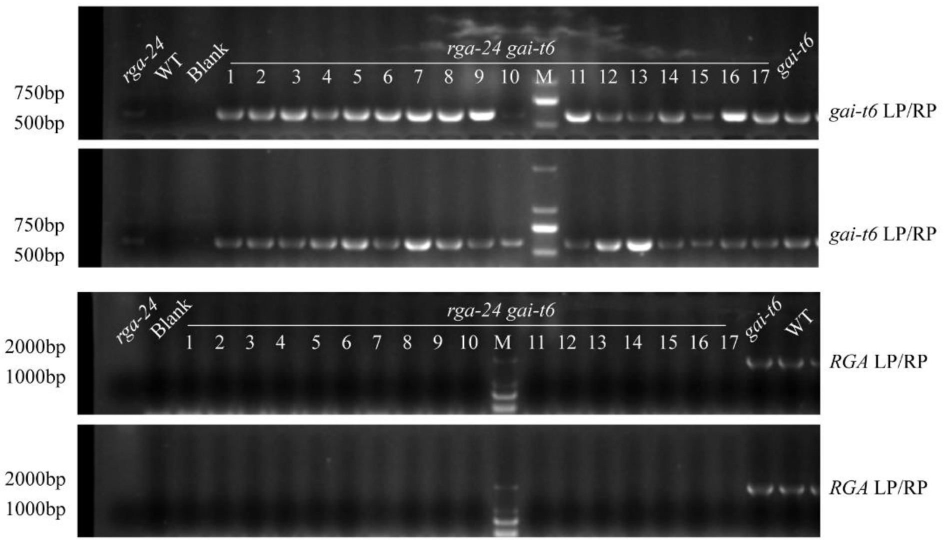

Supplemental Figure S1 Detection of *rga-24 gai-t6* double mutant genotype level. The letter 'M' indicated Maker, 1~17 indicate the number of double mutant.

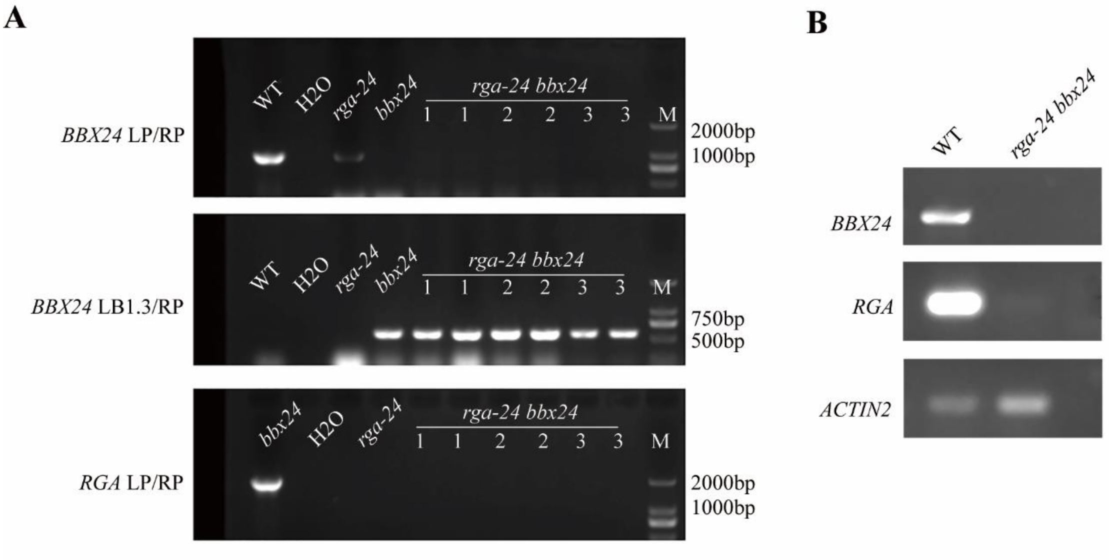

Supplemental Figure S2 Identification of *rga-24 bbx24* homozygous double mutant A. Detection of *rga-24 bbx24* double mutant genotype level. The letter 'M' indicated Maker, 1~3 indicate the number of double mutant. B. Detection of *rga-24 bbx24* double mutant gene expression levels. The *Actin2* gene was as an internal control.

Supplemental Table S1

Gene	Primers	Sequences of primers(5′→3′)
At1g06040	*bbx24*	GACGAGAGGAAGAAGGAGAGC<br>TGAAAGAAATCGTCAACAGCC
—	*LB1.3*	ATTTTGCCGATTTCGGAAC
At2g01570	*RGA*	TTCACATAGAGAAGTCACATGTTCC<br>GTCTTGGTCCGAACATATGTCAT
At1g14920	*gai-t6*	TTCACATAGAGAAGTCACATGTTCC<br>TCGGTACGGGATTTTCGCA
At2g01570	*RGA*	CAAGGTTATCGTGTGGAGGAGAG<br>CAAGCGGAGGTGGTAATGAGTG
At1g14920	*GAI*	CTATGCTCACCGACCTTAATCCT<br>TGGTTAGACGAAGAAGCCGAAT
At1g06040	*BBX24*	TTGCGATATCTGCCAAGAGAAGGC<br>TTCATCGCAGTCCCTGCAAAGC
At5g11260	*HY5*	CCATCAAGCAGCGAGAGGTCATCAA<br>CGCCGATCCAGATTCTCTACCGGAA
At5g13930	*CHS*	ACGTCACGTGTTGAGCGAGTATGG<br>GAGGAACGCTGTGCAAGACGACTG
At5g08640	*FLS*	ATACAGGGAGGTGAATGAA<br>ACACGGCGGATAATAGTT
At5g54060	*UF3GT*	ATCGAATGAATCGTCAAGCATGAG<br>TGAGGGATAGAGATGGTGTGGAAAG
At3g18780	*ACTIN2*	GCTCTTCAGGAGCAATACGAAG<br>GTTGGGATGAACCAGAAGGA

Supplemental Table S2

Y2H Vector construction	*RGA*	CAGTGAATTCCACCCGATGAAGAGAGATCATCACCA<br>TATCGATGCCCACCCTCAGTACGCCGCCGTCGAGAGT
	BBX24	CAGTGAATTCCACCCGATGAAGATACAGTGTGATGT<br>TATCGATGCCCACCCTTAGCCAAGATCAGGGACAA
	HY5	CAGTGAATTCCACCCGATGCAGGAACAAGCGACTAGC<br>TATCGATGCCCACCCTCAAAGGCTTGCATCAGCAT
BiFC Vector construction	*RGA*	CCCAGGCCTACTAGTGGATCCATGAAGATACAGTGTGATGTG<br>ACCCTCGAGGTCGACGGATCCTTAGCCAAGATCAGGGACAAT
	BBX24	TGGCGCGCCACTAGTGGATCCATGCAGGAACAAGCGACTAGC<br>GACAGTACTATCGATGGATCCAAGGCTTGCATCAGCATTAGA
	HY5	TGGCGCGCCACTAGTGGATCCATGCAGGAACAAGCGACTAGC<br>GACAGTACTATCGATGGATCCAAGGCTTGCATCAGCATTAGA
